# Bioactive flavanoids from *Glycosmis arborea*

**DOI:** 10.1186/2191-2858-3-4

**Published:** 2013-03-04

**Authors:** Mohammad Faheem Khan, Nisha Negi, Rajnikant Sharma, Devendra Singh Negi

**Affiliations:** 1 Department of Chemistry, HNB Garhwal University, Srinagar (Garhwal), Uttarakhand 246174, India

**Keywords:** *Glycosmis arborea*, Rutaceae, Flavone C-glycoside, Antifeedant activity, Antimicrobial activity

## Abstract

**Background:**

*Glycosmis* is a genus of evergreen glabrous shrub and distributed all over India. It possesses various medicinal properties and is used in indigenous medicine for cough, rheumatism, anemia, and jaundice. *Glycosmis arborea* is a rich source of alkaloids, terpenoids, coumarins, as well as flavonoids.

**Results:**

The chemical investigation of methanol fraction of the leaves of *G. arborea* led to the isolation of one new flavone C-glycoside along with three known flavanoids, named as 5,7-dihydroxy-2-[4-hydroxy-3-(methoxy methyl) phenyl]-6-C-*β*-d-glucopyranosyl flavone (4), 5,7,4^′^-trihydroxy-3^′^-methoxy flavone (1), 5,4^′^-dihydroxy-3^′^-methoxy-7-*O*-*β*-d-glucupyranosyl flavanone (2), and 5,4^′^-dihydroxy-3^′^-methoxy-7-*O*-(*α*-l-rhamnosyl-(1‴→6‴)-*β*-d-glucopyranosyl) flavanone (3), respectively. The structures of all compounds were elucidated with the help of nuclear magnetic resonance spectrometry. Pure compounds and fractions were evaluated for pest antifeedant and antimicrobial activity.

**Conclusion:**

Four compounds were isolated from the leaves of *G. arborea*. Among them, compound 4 showed significant antimicrobial activity.

## Background

*Glycosmis* is a genus of evergreen glabrous shrub, distributed in warm and temperate regions of the world, and is a rich source of alkaloids and amide; however, terpenoids, coumarins, and flavonoids were also reported [1,2]. Previously, a new carbazole alkaloid, designated as glycoborinine, was isolated from the roots of *Glycosmis arborea*, along with two known alkaloids, carbazole glycozoline and glycozolidine, and two known quinoline alkaloids, *viz.* skimianine and 3-(3^′^,3^′^-dimethylallyl)-4,8-dimethoxy-*N*-methylquinolin-2-one [3]. There are about 60 species in the Indo-Malaysia region, and 7 are found in India. In Uttarakhand, *G. arborea* (Hindi-*Ban Nimbu*, Sanskrit-*Ashvashokta*) grows commonly in Sal and miscellaneous forests of Tarai Bhabher at 600-m heights [4].

**Figure 1:**
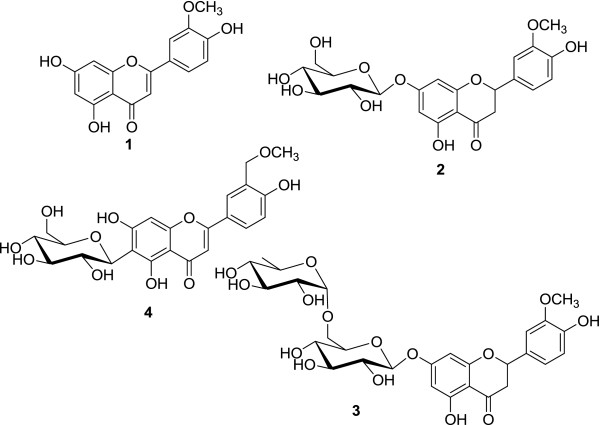
**Chemical structure of isolated compounds from *G. arborea *leaves.**

As a part of our ongoing studies aimed at the phytochemical and pharmacological characterization of this plant, we found that hexane and methanol fractions of the ethanol extract of *G. arborea* leaves showed significant antifeedant and antimicrobial activity. Herein, therefore, we decided to carry out a detailed study to investigate the chemical composition of *G. arborea*. In particular, we report the isolation and characterization of one new flavone C-glycoside (**4**) along with three known compounds (**1** to **3**) (Figure 1) with their antifeedant and antimicrobial activities. The known compounds were identified by using spectroscopic methods including infrared (IR), UV, mass, and 1D and 2D nuclear magnetic resonance (NMR) analysis and also by comparing data already reported in the literature. Methanol fraction yielded one new flavone C-glycoside (**4**) and three known compounds *viz.* 5,7,4^′^-trihydroxy-3^′^-methoxy flavone (**1**) [5], 5,5^′^-dihydroxy-4^′^-methoxy-7-*O*-*β*-d-glucupyranosyl flavanone (**2**) [6], and 5,5^′^-dihydroxy-4^′^-methoxy-7-*O*-(l-rhamnosyl-(1‴→6‴)-*β*-d-glucopyranosyl) flavanone (**3**) [7]. Among these, compounds **1** and **2** have been reported for the first time from *G. arborea*.

## Methods

### Plant material

The *G. arborea* leaves were collected from Rajaji National Park, Rishikesh, Uttarakhand, India during the flowering season and identified by a taxonomist of the Botany Department of HNB Garhwal University Uttarakhand. A voucher specimen is deposited in the Department of Botany, HNB Garhwal University, Uttarakhand.

### Extraction and isolation

Parts of the dried leaves of *G. arborea* (1.8 kg) were air dried, grinded, and refluxed with 90% ethanol. The total ethanol extract was concentrated under reduced pressure at a temperature below 50°C to a dark green viscous mass coded as F001 (120 g) that was partitioned with hexane (F003) (21 g) and *n*-butanol F004 (88 g). The *n*-butanol soluble layer was then successfully fractionated into chloroform (4 g) (F005), ethyl acetate (19 g) (F006), and methanol soluble fraction (63 g) (F007). The methanol soluble fraction (F007) after removal of the solvent was chromatographed over silica gel (800 g) and eluted with mixtures of CHCl_3_/MeOH as eluents to give four fractions A1 to A4 (9:1, 88:12, 85:15, 82:18). Fraction A2 (2.4 g) was rechromatographed on silica gel with CHCl_3_/MeOH mixtures (9:1, 85:15) and yielded compounds **1** (70 mg), **2** (90 mg), **3** (46 mg), and **4** (36 mg).

#### 5,7,4^′^-trihydroxy-3^′^-methoxy flavone (1, C_16_H_12_O_6_)

M.p. 287°C to 288°C; UV (MeOH) *λ*_max_ nm: 214, 241, 268, 348; IR (KBr) *ν*_max_ cm^−1^: 3431, 1634, 1594, 1382, 1351, 1078, 770. ESIMS (C_16_H_12_O_6_) *m/z*: 300 [M]^+^, 285 [M-CH_3_]^+^, 272 [M-CO]^+^, 257 [M-43]^+^, 241, 242, 215, 204, 193, 176, 152 [A_1_]^+^, 148 [B_1_]^+^, 136, 124 [A_1_-28]^+^ 107, 105. ^1^H NMR (400 MHz, DMSO-*d*_*6*_): *δ* ppm 6.62 (1H, s, H-3) 6.05 (1H, d, *J* = 2.0 Hz, H-6), 6.38 (1H, d, *J* = 2.0 Hz, H-8), 7.63 (1H, d, *J* = 2.4 Hz, H-2^′^) 6.87 (1H, d, *J* = 9.0 Hz, H-5), 7.78 (1H, dd, *J* = 2.4, 9.0 Hz, H-6) 3.86 (3H, brd s, OCH_3_). ^13^C NMR (100 MHz, DMSO-*d6*): *δ* ppm 161.02 (C-2) 106.32 (C-3), 182.2 (C-4) 160.9 (C-5), 98.7 (C-6), 164.2 (C-7), 93.9 (C-8), 103.6 (C-4a), 157.1 (C-8a), 124.1 (C-1^′^), 115.6 (C-2^′^), 148.4 (C-3^′^), 145.2 (C-4^′^), 117.0 (C-5^′^), 122.3 (C-6^′^), 56.2 (OCH_3_).

#### 5,4^′^-dihydroxy-3^′^-methoxy-7-O-β-d-glucupyranosyl flavanone (2, C_22_H_24_O_11_)

M.p. 264°C; UV (MeOH) *λ*_max_ nm: 295, 328. IR (KBr) *ν*_max_ cm^−1^: 3423, 1620, 1594, 1351, 1071, 1012, 974, 921. ESIMS (% int.) (C_22_H_24_O_11_) *m/z*: 464 [M]^+^ (70), 449 [M-CH_3_]^+^ (3), 416 [M-OCH_3_-OH]^+^ (17), 381 (88), 353 (93), 302 [M-Glu]^+^ (4), 301 (4), 203 (6). ^1^H NMR (400 MHz, DMSO-*d*_*6*_): *δ* ppm 12.01 (1H, s, OH-5), 9.09 (1H, s, OH-5^′^), 5.47 (1H, dd, *J* = 6.2, 9.2 Hz, H-2) 2.8 (1H, dd, *J* = 12.4 Hz, H-3a), 3.29 (1H, dd, *J* = 13.0, 17.2 Hz, H-3b), 6.13 (1H, d, *J* = 2.4 Hz, H-6), 6.10 (1H, d, *J* = 2.4 Hz, H-8), 6.92 (1H, dd, *J* = 3.2, 8.0 Hz, H-2^′^), 6.89 (1H, d, *J* = 8.4 Hz, H-3^′^), 6.92 (1H, d, *J* = 3.2 Hz, H-6), 4.67 (1H, d, *J* = 7.6 Hz, H-1″), 3.42-3.82 (5H, m, sugar), 3.76 (3H, brd s, OCH_3_). ^13^C NMR (100 MHz, DMSO-*d*_*6*_): *δ* ppm 78.77 (C-2), 42.4 (C-3), 197.44 (C-4), 165.52 (C-5), 96.3 (C-6), 163.43 (C-7), 95.51 (C-8), 103.69 (C-4a), 162.88 (C-8a), 131.27 (C-1^′^), 112.2 (C-2^′^), 118.33 (C-3^′^), 148.36 (C-4^′^), 146.83 (C-5^′^), 114 (C-6^′^), 100.99 (C-1″), 71 to 76 (C-2″-C-5″), 66.41 (C-6″), 56.05 (OCH_3_).

#### 5,4^′^-dihydroxy-3^′^-methoxy-7-O-(α-l-rhamnosyl-(1‴→6″)-β-d-glucopyranosyl) flavanone (3, C_28_H_34_O_15_)

UV (MeOH) *λ*_max_ nm: 293, 326. IR (KBr) *ν*_max_ cm^−1^: 3472 (OH), 1630, 1602 (aromatic), 1521, 1352, 1092, 815. ESIMS (C_28_H_34_O_15_) (% int.) *m/z*: 610 [M]^+^, 633 [M + Na]^+^ (54), 595 (2), 551 (5), 507 (7), 463 (7), 388 (77), 364 (93), 338 (70), 306 (50), 233 (35), 179 (43), 151 (16). ^1^H NMR (400 MHz, C_5_H_5_N-*d*_*5*_): *δ* ppm 12.24 (1H, s, OH-5), 8.84 (1H, s, OH-5^′^), 5.45 (1H, dd, *J* = 6.2, 9.6 Hz, H-2), 2.85 (1H, dd, *J* = 12.4 Hz, H-3a), 3.22 (1H, dd, *J* = 13.0, 17.2 Hz, H-3b), 6.49 (1H, d, *J* = 2.1 Hz, H-6), 6.60 (1H, d, *J* = 2.1 Hz, H-8), 7.11 (1H, dd, *J* = 2.1, 6.3 Hz, H-2^′^), 6.96 (1H, d, *J* = 6.3 Hz, H-3^′^), 7.51 (1H, d, *J* = 2.1 Hz, H-6), 5.69 (1H, d, *J* = 7.6 Hz, H-1″), 4.26 to 4.66 (5H, m, H-2″-H-6″), 5.43 (1H, d, *J* = 3.0 Hz, H-1‴), 4.14 to 4.60 (4H, m, H-2‴-H-5‴), 1.57 (3H, d, *J* = 5.7 Hz, H-6‴), 3.71 (3H, brd s, OCH_3_). ^13^C NMR (100 MHz, C_5_H_5_N-*d*_*5*_): *δ* ppm 79.50 (C-2), 43.16 (C-3), 197.1 (C-4), 166.48 (C-5), 96.44 (C-6), 164.08 (C-7), 97.32 (C-8), 104.34 (C-4a), 163.49 (C-8a), 132.15 (C-1^′^), 118.49 (C-2^′^), 112.31 (C-3^′^), 148.43 (C-4^′^), 149.12(C-5^′^), 115.32 (C-6^′^), 102.51 (C-1″), 72.79 to 78.45 (C-2″, C-5″), 67.37 (C-6″), 101.55 (C-1‴), 69.83 to 77.24 (C-2″, C-5‴), 18.61 (C-6‴, methyl), 55.89 (OCH_3_).

#### 5,7-dihydroxy-2-[4-hydroxy-3-(methoxymethyl)phenyl]-6-C-β-d-glucopyranosyl flavone (4, C_23_H_24_O_11_)

UV (MeOH) *λ*_max_ nm: 208, 306. IR (KBr) *ν*_max_ cm^−1^: 3437, 1660, 1590, 1350, 1074, 901, 836. EIMS (C_23_H_24_O_11_) (% int.) *m/z*: 493 [M + OH]^+^ (18), 478 [M + 2H]^+^ (11), 448 [M-CO]^+^ (14), 455 (94), 444 (11), 433 (9), 413 (22), 301 (4), 260 (3), 203 (2), 136 (2). ^1^H NMR (400 MHz, DMSO-*d*_*6*_) and ^13^C NMR (100 MHz, DMSO-*d*_*6*_) *δ* (see Table 1).

**Table 1:** **NMR spectroscopic (400 MHz) data of compound 4 in DMSO-*d***_***6***_

**Positions**	^**1**^**H ( *J *in Hz)**	^**13**^**C**	**HSQC**	**HMBC**	
				^***2***^***J***	^***3***^***J***
2	-	161.51	qC		
3	6.77 (s)	103.46	CH	C-4	C-4a,1^′^,5^′^
4	-	182.47	qC		
5	-	162.97	qC		
6	-	104.97	qC		
7	-	156.36	qC		
8	6.26 (s)	98.50	CH	C-8a	C-6, 2
4a	-	104.38	qC		
8a	-	160.75	qC		
1^′^	-	121.98	qC		
2^′^	8.02 (d, *J* = 2.4, 8.4)	129.34	CH		C-5^′^, 8a
3^′^	6.88 (d, *J* = 8.4)	116.17	CH		C-1^′^
4^′^	-	151.42	qC		
5^′^	-	164.30	qC		
6^′^	6.91 (d, *J* = 2.4)	116.17	CH	C-1^′^	C-3^′^
1″	4.65 (d, *J* = 9.9)	73.74	CH	C-6, 2″	C-3″, 5″, 5, 7
2″	3.82 (dd, *J* = 9.2,9.6)	70.86	CH	C-1″	
3″	3.24 (t, *J* = 9.2)	79.01	CH		
4″	3.34 (t, *J* = 9.2)	71.18	CH		
5″	3.22 (ddd, *J* = 5.0, 9.2)	82.22	CH		C-1″
6″	3.75 (d, *J* = 11.6)	61.65	CH_2_	C-5″	
	3.52 (dd, *J* = 6.0,12.0)				
1‴	3.17 s	48.97	CH_2_		
OCH_3_	3.89	56.8	CH_3_	C-4^′^	
OH-5	13.15				

### Antifeedant assay

The antifeedant activity of the extracts against the polyphagous pest *Spodoptera litura* was tested using the leaf dip method [7]. Five percent concentrations of each extract were prepared by dissolving extracts in a small quantity of ethanol and diluting in water containing 0.05% Triton X-100. The leaf discs of about 5 cm^2^ were prepared out of castor leaf (*Ricinus communis* L.) and were dipped for 30 s in an extract or compound separately. The leaf discs dipped only in water containing 0.05% Triton X-100 were used as controls. The leaf discs were air dried, and on each treated leaf disc, 10 larvae of *S. litura* (1 day old) were released. Three replications were maintained for each extract. Larval weight was taken after 4 days of treatment. Antifeedant activity of fractions and the purified compounds were tested against the polyphagous crop pest *S. litura* (Table 2).

**Table 2:** **Pest antifeedant activity of *G. arborea *fractions against *S. litura *L**

**Fractions**	**Percent feeding index (PFI) 2.5 (μg/cm**^**2**^**)**
Hexane	46.71 ± 4.07
Methanol	50.21 ± 5.21

A concentration of 0.05% Triton X-100 was used as control.

### Antibacterial assay

The *in vitro* antibacterial activity was tested by the disc diffusion method [8] using pathogenic strains of *Agrobacterium tumifaciens*, *Pseudomonas syringae*, and *Pectobacterium. carotovorum*. Concentrations of 200 and 500 μg/disc of compounds were impregnated on the discs. These discs were placed on the surface of the agar plates already inoculated with pathogenic bacteria. The plates were incubated at 37°C and examined at 48 h for zone of inhibition, if any, around the discs. Gentamicin was used in the assay as a standard control drug. An additional control disc without any sample but impregnated with an equivalent amount of solvent (DMSO) was also used in the assay. The result of antibacterial activity indicated that methanol fraction and compound **4** exhibited a mild to moderate activity (Table 3).

**Table 3:** **Antibacterial activity of *G. arborea *fraction and isolated compound against plant bacterial pathogens**

**Particular**	**Concentration (μg/disc)**	**Zone of inhibition (in mm)**		
		***Agrobacterium tumifaciens***	***Pseudomonas syringae***	***Pectobacterium carotovorum***
Methanol fraction	200	12	-	14
	500	16	6	18
Compound **4**	200	9	-	-
	500	11	-	-

Gentamicin was used as a standard control drug.

## Results and discussion

### Chemistry

The repeated chromatography of methanol fraction of the leaves of *G. arborea* led to the isolation of four flavonoids by gradient elution with the CHCl_3_/MeOH mixture of increasing polarity. Compound **4** was isolated as a yellow solid which was further crystallized in acetone. It had the composition C_23_H_24_O_11_ (*m*/*z* = 476) as derived from the positive mode of electrospray ionization mass spectrometry (ESIMS) analysis. A positive Shinoda test and color reaction with ferric chloride suggested the presence of free phenolic hydroxyl groups. The UV spectrum exhibited absorption maxima at 208 and 306 nm which are characteristic of a flavone skeleton [9]. Its IR spectrum showed the presence of a hydroxyl group at 3,437 cm^−1^ and a chelated carbon at 1,660 cm^−1^ (*γ* pyrone nucleus) along with other absorption bands at 1,590 and 838 cm^−1^, a characteristic of an aromatic nucleus.

The ^1^H NMR spectrum showed the signals typical for flavone moiety. A double doublet at *δ* 8.02 (*J* = 2.4, 8.4 Hz) and doublet at 6.88 (*J* = 2.4 Hz) and in the aromatic region with meta-coupling were assigned to H-2^′^ and H-6^′^. Another doublet at *δ* 7.92 (*J* = 8.4 Hz) was attributed to H-3^′^ proton. Two sharp singlets at *δ* 6.77 and 6.26 were ascribed as H-3 and H-8, respectively. The downfield chemical shift at *δ* 13.15 was assigned to the hydroxyl proton of OH-5. A sharp peak resonated at *δ* 3.8 due to the methoxy proton. ^1^H spectra showed upfield signal at *δ* 3.15 for two protons attributed to aliphatic methylene H-1‴. The ^13^C NMR spectra showed the signals for 23 carbons which were differentiated into 10 methines, 2 methylenes, 1 methyl, and 10 quaternary carbons on the basis of distortionless enhancement by polarization transfer (DEPT; 90 and 135) experiments. DEPT 135 revealed the presence of two methylene carbons in a molecule resonating at *δ* 61.65 and 48.97 assigned to C-6^′^ and C-1‴. From ^1^H spectra, two doublets of one proton resonated at *δ* 4.65 and 3.75 assigned to H-1″ (*J* = 9.9 Hz, anomeric) and H-6″a. Two doublet of doublets of one proton at *δ* 3.82 (*J* = 9.2, 9.9 Hz) and 3.52 (*J* = 12 Hz) were attributed to H-2″ and H-6″b. A multiplet at *δ* 3.22 to 3.34 showed for the remaining three sugar protons.

**Figure 2:**
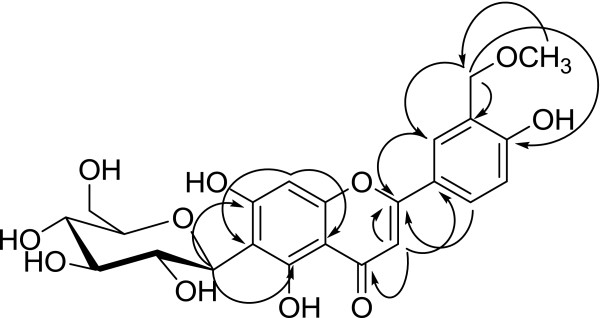
Selected long-range HMBC correlation of compound 4.

The exact proton and carbon assignments were made by a combination of 2D NMR experiments such as COSY, heteronuclear single-quantum correlation (HSQC), and heteronuclear multiple bond correlation (HMBC). The position of sugar was confirmed by HMBC long-range correlation in which anomeric H-1″ showed long-range coupling with 162.97 (C-5), 104.97 (C-6), and 156.3 (C-7), suggesting the position of sugar at C-6 of aromatic ring B (Figure 2). The double bond position was confirmed by coupling of a proton singlet (*δ* 6.77) with 182.47 (C-4) and 121.98 (C-1^′^), suggesting that it must be placed at C-3 and assigned as H-3. The 5-hydroxy flavone skeleton was assigned on the basis of ^13^C NMR data which showed C-4 resonance at *δ* 182.47, characteristic of 5-hydroxy flavone [10,11]. The signal resonating at *δ* 56.8 was due to methoxy carbon. The upfield appearance of anomeric carbon (73.74) and proton (4.65) as compared to those of aromatic O-glycoside data and anomeric proton correlation with C-5, C-6, and C-7 in the long-range HMBC experiment exhibited its C-glycosidic nature which was confirmed by its resistance to acidic hydrolysis [12,13]. The remaining HMBC correlations are given in Table 1.

The structure was supported by the mass spectroscopic studies, which showed molecular ion peak at 478 *m/z* [M^+^+2H]. Fragment at 445 *m/z* was due to loss of carbonyl [M^+^-CO] and further at 433 *m/z* was due to loss of the methyl group [M^+^-CO-CH_3_]. A higher percentage of fragmentation also appeared at 455 *m/z* [M^+^-H_2_O-3H]. Thus, compound **4** was unambiguously identified as 5,7-dihydroxy-2-[4-hydroxy-3-(methoxymethyl) phenyl]-6-C-*β*-d-glucopyranosyl flavone, a new flavone C-glycoside named as Arboreaside.

### Biological studies

#### Antifeedant activity

All the fractions and compounds were tested for antifeedant activity. Among them, the hexane and methanol fractions showed significant antifeedant action against *S. litura* L. In a dual-choice leaf disc method, hexane and methanol fractions were tested for pesticidal potential. The hexane fraction showed a percent feeding index (PFI) of 46.71 ± 4.07, while the methanol fraction showed a PFI of 50.21 ± 5.01 as given in Table 2.

#### Antibacterial activity

All the fractions and compounds were also tested for antibacterial activity. The methanol fraction and compound **4** showed antimicrobial activity against the plant bacterial pathogens *A. tumifaciens*, *P. syringae*, and *P. carotovorum.* It was found that moderate inhibitory activities were observed against *A. tumifaciens* and *P. carotovorum* at a concentration of 200 μg, whereas *P. syringae* was found to be fatal at 500 μg. Compound **4** showed moderate inhibition against *A. tumifaciens* as shown in the Table 3*.*

### Experimental

All melting points are uncorrected and were taken in open capillaries. The UV spectra were recorded on a PerkinElmer Lambda 15 UV/VIS spectrophotometer (PerkinElmer, Waltham, MA, USA) in methanol as blank. IR spectra were recorded on a PerkinElmer Infrared 15 in KBr pellets and are expressed per centimeter. The ^1^H and ^13^C NMR were scanned on a Bruker AVANCE 400 MHz (Bruker Corporation, Billerica, MA, USA) at C_5_D_5_N-*d*_*5*_, DMSO-*d*_*6*_, CD_3_OD, and CDCl_3_ at 400, 300, and 100 MHz with TMS as internal reference. Proton-detected heteronuclear correlations were measured using HMQC (optimized for *J*_HC_ =14.5 Hz) and HMBC (optimized for *J*_HC_ =7 Hz). Mass spectra were recorded on a Micromass Quattro II (Micromass UK Ltd., Manchester, UK) at 70 eV for ESIMS. Column chromatography was carried out using silica gel (60 to 120 mesh, Qualigen (Carlsbad, CA, USA)/Merck (Whitehouse Station, NJ, USA)). Thin layer chromatography was carried out over plates made of silica gel G of Qualigen/Merck.

## Conclusion

In conclusion, the present paper has shown the isolation and structure elucidation of one new flavone C-glycoside (**4**) along with three known compounds (**1** to **3**) from methanol fraction of *G. arborea* leaves. With regard to bioactivity, all fractions and isolated compounds were evaluated for antifeedant and antibacterial activity. Among them, hexane and methanol fractions showed antifeedant activity, whereas methanol fraction and compound **4** showed significant antibacterial activity.

## Competing interests

The authors declare that they have no competing interests.
